# The Transactions of the Provincial Medical and Surgical Association. Vol. IX

**Published:** 1841-07-01

**Authors:** 


The Transactions of the Provincial Medical and Surgical Association. Instituted 1832. Vol. IX. London: John Churchill, 1841.

Our Provincial Brethren continue to display the same zeal, the same steadiness of purpose, and the same information, as when they first began to publish their Transactions. These volumes are highly useful—they keep alive a spirit of observation, and acquaint the professional world with many valuable facts which would otherwise be unrecorded.

The contents of the present volume consist of—I. Proceedings of the Association at Southampton,—which comprise, Address by Dr. Jeffreys—Address by Dr. Steed—Report of the Council—Thackeray Prize—Parochial Medical Relief—Vaccination Section—Medical Reform—Report on Empiricism—Report of the Benevolent Committee—Medical and Surgical Papers and Cases.

II. Retrospective Addresses.—The Retrospective Address in Surgery, delivered at the Eighth Anniversary Meeting of the Provincial Medical and Surgical Association, held at Southampton, July 22nd and 23rd, 1840. By A. T. S. Dodd, Esq., Surgeon to the Infirmary, Chichester.—The Retrospective Address, delivered at the Eighth Anniversary Meeting of the Provincial Medical and Surgical Association, held at Southampton, July 22nd and 23rd, 1840. By Roger W. Scott, M.D., Physician to the South Dispensary, Liverpool.

III. Medical Topography.—Medical Topography of Shrewsbury and its Neighbourhood. By T. Ogier Ward, M.D., Shrewsbury.—Statistics of Rheumatism. By Edmund Lyon, M.D., Physician to the Manchester Royal Infirmary, Dispensary, &c.

IV. Essays and Cases.—Observations relating to Injuries of the Skull and Brain, in which the use of the Trephine is considered. By John M. Banner, Esq., Surgeon to the Northern Hospital, Liverpool, and Lecturer on Surgery at the Medical School of the Liverpool Royal Institution.—On the Moveable Cartilaginous Bodies found in the Synovial and Serous Cavities. By James Macartney, M.D. F.R.S., Honorary Member of the Provincial Medical and Surgical Association, &c.—A Case of Amputation of the greater part of the Lower Jaw. By A. T. S. Dodd, Esq. Surgeon to the Infirmary, Chichester.—Cases of Abscess forming within the Pelvis after Labour, with Observations. By Thomas W. Wainwright, Esq. Surgeon to the Liverpool Northern Hospital.—Cases illustrative of the Pathology of Biliary Concretions. By George Mallett, Esq., Surgeon, Bolton-le-Moors.—On the Influence of the Depressing Passions on the Health and on Disease. By J. K. Walker, M.D. one of the Physicians to the Infirmary, Huddersfield.

V. Infirmary Reports and Medical Statistics.—A Report of the Out-Patients, attended by F. Ryland, Esq., at the Birmingham Town Infirmary, between the 25th December, 1837, and the 12th December, 1839.—A Report of the Cases attended at the Birmingham Eye Infirmary during the Years 1838 and 1839. By Richard Middlemore, Esq. Surgeon to the Infirmary.—Suggestions as to the form of Register for Hospitals, Dispensaries, and Private Practice. By Charles Cowan, M.D., Physician to the Royal Berkshire Hospital and Reading Dispensary, Fellow of the London Statistical Society, and Member of the "Société Medicale d'Observation" of Paris.

The Retrospective Addresses we cannot praise too highly. They severally offer a valuable epitome of what has been done in medicine and surgery during the last twelve months, and will be read with the same interest which must have waited on their delivery.

THE MEDICAL TOPOGRAPHY OF SHREWSBURY is cleverly handled by Dr. Ward.

We can only notice one or two points connected with it. The first of these is the physical character of the inhabitants, who are either agricultural, mining, or townsmen. The recruiting officer, an authority, declares that the men enlisted in this district are quite equal, if not superior, to the ordinary average. This however applies to the agricultural and town population only; for the inhabitants of the coal district are a short, stunted race, broad-chested and powerful upwards, but weak in the legs and very subject to deformities of the toes, that unfit them for marching. This weakness of their lower limbs is owing to the constrained positions of sitting, kneeling, or lying on the side, in which they work in extracting the coal and ironstone; and these positions are rendered so habitual to the colliers, that they generally prefer squatting on their hams to any other posture, either at home, or when congregated in the streets, where they may be seen sitting in rows with their backs against the wall. The miners work in less constrained postures in following the veins of ore, and hence they do not differ in figure from other men. Except the dreadful accidents from explosion, &c. to which both colliers and miners are exposed, their occupation does not seem to produce any directly prejudicial effects upon either of them. False melanosis of the lungs, from the inhalation of particles of coal, and of the smoke of gunpowder in the pits, is almost unknown in the coal districts. Some of the lead miners however have suffered from colica pictonum. Independently of disease, the whole class of colliers, iron-founders, and lead-miners, are subject to premature old age, from the intense heat which some, and the hard labour which all undergo.

*Variations of the Population.*—These shew the influence of employment upon population. Where the former is rife, there is an influx of labourers—when scarce, an emigration of them. This important and natural fact must be recollected at the present moment when the operation of the corn-laws is so engrossing a question.

In the first decennial period, the population of Shrewsbury increased only as much as would result from the average excess of births over deaths; in the second, half as much more; and in the third, but little more than half. The county population, on the contrary, increased a third more in the first period, only half in the second, and two-thirds of the average in the third. The mining parishes increase their numbers by a fourth only of the average in the first decade, by a fourth more than that sum in the second, and by double the average in the third.

A reference to the third table will explain in some degree the causes of this fluctuation, by shewing that it depends in a considerable degree upon the migrations of the male population in search of employment; thus, at the last census, nearly nine per cent. of the males had quitted Shrewsbury, while a number of men, equal to five per cent. of the inhabitants, had immigrated into the mining parishes; the county population experiencing little influx of males, but rather lessening by the emigration both of males and females.

Dr. Ward does not know what peculiar causes operated to increase the town population during the second decade, but the falling off in the mining district was doubtless owing to the great encouragement given to agriculture by the increased price of wheat (nearly forty per cent.) above what it bore in the previous ten years; for we observe that, though the mining population decreased, the whole county increased more than the average. The second decade, embracing some years of peace, gave a stimulus to manufactures, and not only prevented the emigration of the miners, but produced a reflux of the tide from the agricultural part of the county sufficient to restore the average, and even more; and this stream has continued to flow during the third decade with augmented velocity, which, to judge from the present state and prospects of the iron trade, there is every reason to believe it still retains.

*Great Prevalence of Insanity.*—An examination of a table of the insane presents us, observes Dr. Ward, with a very unsatisfactory view of the intellectual state of the county of Salop, whether we compare it with the adjoining ones, or with such as exhibit an approximation to it in any of the points of comparison mentioned. In the first place, the number of the insane in Salop is exactly the same as in the adjoining county of Stafford, but the proportion to the population is nearly twice as great, and the proportion of idiots almost twenty per cent. more; and we observe the same inferiority if we take a county partly mining and partly agricultural, as Northumberland; or one wholly agricultural, as Sussex. In the next class, where the proportions are similar of the insane and the population, the proportion of idiots averages eleven per cent. less than in Salop. Dr. Ward justly remarks that the prevalence of idiotcy may be taken in some degree as the measure of the intellectual degradation of a country, though doubtless climatorial influence, as in the canton of the Vallais, in Switzerland, has considerable effect in the production of this form of insanity. In the third class we see a close approximation to the state of Salop, if we take into consideration both the proportion of insane to the population and the per centage of idiots, except in Buckinghamshire, which, it is remarkable, was, previous to the passing of the new Poor Law, in the worst state, with regard to pauperism, of any county in the kingdom. All these counties it may be observed are agricultural. Lastly, if we compare Salop with the general average of England, and of England and Wales, we find it inferior to both united, though superior to Wales alone, to which it thus rather closely approaches, not only in climate and soil, but also in intellectual character. It is worthy of remark that the proportion of idiots is the highest in three of the most mountainous counties of Wales, viz., Carnarvon 68.4, Denbigh 75.9, and Merioneth 82. Having thus exhibited the dark side of the picture, it is but right to take a more favourable view, and to state that there are few counties in the kingdom that can boast of a longer list of worthies in the sciences, arts, or arms, than Shropshire.

### STATISTICS OF RHEUMATISM.

Dr. Lyon, of the Manchester Royal Infirmary, contributes these.

It appears that the aggregate of rheumatic cases in ten years was 291 out of 3561 *home-patients*, or a little more than 8 per cent.; that 103 of these were chronic, and 188 more or less acute, making the ratio of the latter something more than 5¼ per cent. (5.28.)

On referring to the monthly accounts it will be seen that they are highest, both absolutely and relatively, in April and May, and lowest in August and September,—being, in April, 35 out of 332, or 10.54 per cent.; in May, 40 out of 329, or 12 per cent.; in August, 13 out of 282, or 4.6 per cent.: and in September, 18 out of 324, or 5.5 per cent.:—or, taking the acute and subacute cases only, 6.9 and 7.9 per cent. in April and May, and only 3.19 and 3.7 per cent. in August and September. Next to these, the months of February, March, and December, exhibit the smallest proportion of rheumatic cases.

If we now advert to the influence of *sex*, it will be seen that, of the whole number of rheumatic cases recorded in the table, the number of males and females was nearly equal,—145 of the former, and 146 of the latter; or, throwing out the chronic cases, 96 males, and 92 females,—a very different result from what is observed in the kindred disorder, gout. Nevertheless the absolute numbers must not be taken as a true measure of the liability of the two sexes to this affection; for, the total number of males admitted being 1439, and of females 2122, calculation will show a relative excess of rheumatic males over females, including the chronic cases, in the ratio of 10. to 6.88 per cent.; and, excluding them, in the ratio of 6.67 to 4.33 per cent.; or, in either case, very nearly as 3 to 2. It may also be remarked, with respect to the acute and subacute cases, that whilst, in January, August, September, and December, there is a slight excess of females, in April the males are as 10.44 to 4.5, and in May as 11.59 to 5.23.

After furnishing a very carefully compiled table, Dr. Lyon proceeds:—

"It may be inferred from this series of observations that the tenderness and delicacy of habit belonging to young females render them less liable than youths of the other sex to rheumatic attacks, and that women who have passed the meridian of life become even more liable to such attacks than men of the same age; whilst in both sexes the middle period of life is most favourable to the production of this disease. But if women be, on the whole, less exposed than men to rheumatism, they appear to be more frequently cut off by its attacks; for, of four *deaths* recorded in the 188 cases, three took place in women at the ages of fifty-six, thirty-seven, and nineteen years; and I would attribute this greater fatality in *them* to their greater susceptibility of a particular complication of the disease, which does not appear to have been duly noticed by systematic writers. The complication in question consists of a pulmonary affection—most commonly in the form of bronchitis—coexistent with the inflammation of the large joints, sometimes superseding it, and occasionally proving fatal. I have not the means of stating accurately the proportion which these complicated cases bear to the whole number; but, on a rough estimate, I would say that not less than a fourth part of my cases have been of this character, more or less strongly marked." 343.

### Observations relating to Injuries of the Skull and Brain, in which the Use of the Trephine is Considered. By John M. Banner, Surgeon to the Northern Hospital, Liverpool, and Lecturer on Surgery, at the Medical School of the Liverpool Royal Institution.

This is a long and valuable paper. We shall notice some of its prominent points.

After relating seven cases, Mr. Banner observes—" that so long as a patient can be roused to a state of consciousness, or so long as voluntary motion has not ceased, a surgeon would be very wrong in using the trephine, unless some peculiarity in the case rendered it necessary, which we will notice hereafter. In the cases of Hoy and Parkinson a discharge of blood took place from the ear, which may be considered an almost exclusive proof that the fracture extends through the petrous portion of the temporal bone; and where this is observed, I consider it a strong reason against the operation, even in those cases where the symptoms are sufficiently urgent to justify it if this symptom were not present. I would not be understood as considering the fact of a fracture extending through the temporal bone as a fatal consequence; on the contrary, I could relate several cases where this symptom existed, as in those already related, where recovery took place. I would however insist that in urgent cases, where there is fracture, for instance, of the parietal bone, and hæmorrhage from the ear, and compression so great as to destroy the functions of the brain, the operation would be unjustifiable; for in all such cases I have invariably found blood effused at the base of the skull, and often severe injury to the brain itself. It may be said 'but why not give the patient even this small chance, for without it he must die?' I would suggest that an operation should not be performed in this nor any other case unless there is a *fair prospect of success.*"

Mr. Banner details seven cases of *Laceration of the Brain.* Mr. Banner remarks upon them:—"The six last cases have been selected for the purpose of drawing attention to the circumstance that in each of them existed *convulsive action,* and in each there was *laceration of the brain.* If it could be proved that in the majority of cases of *compression of the brain,* where laceration of that organ existed, convulsions or convulsive twitchings were a consequence, it would render our diagnosis easier; we might conclude that in a large majority of such cases the blood would be effused beneath the membranes of the brain, and thus render the operation of trephining the skull inapplicable. Cases, however, do occur, proving that convulsions, when attendant on severe injury of the brain, are not always a fatal symptom; this is instanced, in cases of concussion only, where the patients recover; nevertheless this is not a reason why there may not exist laceration of the brain. The cases I am anxious to draw attention to are those where the compression is so great as to suggest an operation, but where convulsive motions are present, and probably prove laceration and internal hæmorrhage. I am bound, however, to confess that two cases have occurred within the last year at the North Hospital where, on dissection, we found laceration of the brain, and in which cases the convulsive motions were not present. In these cases there was an immediate destruction of almost all the powers, and death took place soon after their admission." He adds,—there are a vast number of cases recorded where there has been laceration of the brain; and the symptom of *convulsive motions* is mentioned in very few of them.

We must confess that we should doubt the justice of the opinion, that convulsions are any decisive evidence of laceration of the brain. When we reflect on the variety of causes that give rise to convulsions, we must hesitate to admit the necessity for so exclusive and structural a lesion as laceration.

*Ecchymosis of the Eye-lids.*—"Several cases have been admitted into the Hospital where *ecchymosis* of the eye-lids has taken place after a severe injury of the head, often coming on at a period of some hours after the accident; the appearance is very peculiar, the cellular membrane of the lids appears filled with blood, and looks livid and swollen. I have observed that where this appearance has existed in connexion with compression of the brain, in cases which have terminated fatally, that a fracture has taken place in the roof of the orbit, usually extending to the basis of the cranium. I have seen this appearance so often in connexion with a fracture through the orbit, that I consider it an almost sure diagnostic mark of this injury; and where this fracture exists I have observed that there has been effusion of blood at the basis of the cranium, so situated as to render relief from the trephine quite beyond all hope or expectation." 379.

*Perforation of the Skull with a Pointed Instrument.*—Mr. Banner asks if we may not include cases of this kind among those which require the operation of trephining. He believes that an unfavourable termination results in almost every case which is left unassisted by an operation; it is scarcely necessary to state that an almost universal consequence of such an accident is a fracture and depression of the inner table of the cranium, spicula of bone being often chipped off; in either instance the membranes or brain subsequently suffer. Where the perforation is large, he apprehends there cannot be much difficulty in ascertaining the fact of there being depression of the inner table of the skull; not so, however, where the opening is small; in nearly every case which he has met with, the aperture has been so small as to cause great difficulty and doubt as to the extent of injury; this difficulty has been considered by some writers as a reason why the operation of trephining the skull is improper. Experience, however, proves the contrary; the many recorded cases corroborate this opinion. He conceives that when we are called early to such an accident the operation would be justifiable, even though there were no urgent symptoms present. Authentic records prove that in nearly every case of perforated skull the inner table is chipped off, and produces inflammation and suppuration; the matter, as before stated, usually forming beneath the membranes, in the substance of the brain: if it were otherwise, we should not be acting correctly in having recourse to the operation before urgent symptoms were present.

The following are Mr. Banner's sentiments on a question of great interest and of much difficulty—whether we should or should not trephine in a case of compound fracture of the cranium with depression, but *without* "symptoms."

"Injuries of the head, in which there is a wound of the scalp, and fracture of the skull, with depression of bone, without urgent symptoms of pressure on brain, ought not to be interfered with, unless the bone is much comminuted, or where the fractured portion is isolated; in which instances we are called upon to render our aid, by using the trephine. Depression of the cranium may remain in many instances without producing any bad consequences, where it is unattended with symptoms of compression. Again, there are numerous instances where such an injury has been followed by extensive mischief. Suppuration may take place on the surface of the *dura mater*; this, however, cannot happen without our being made aware of it by the sympathy usually attendant on the formation of pus on the brain being present. Many eminent surgeons are of opinion that the extent of the depression should guide us in our determination with regard to the trephine: for instance, where the depression is considerable, that the trephine should be applied; where it is slight, it should be avoided; and this, too, where the scalp may not have received any wound. This rule however affords us no assistance with respect to the greater danger arising from the chance of suppuration between the bone and the *dura mater*; this being as likely to occur where the depression is small as where it is large.

Sir Astley Cooper has stated, in his Lectures on Surgery, that there is a great difference as to the danger of inflammation and suppuration of the membranes of the brain between those cases in which the fracture and depression is complicated, with a wound of the scalp, and those in which the soft parts are uninjured; such mischief being much more liable to occur in cases of the first kind than in those of the second; and on these grounds he recommends that, where the complication exists, *we should not hesitate to apply the trephine*; and on the other hand that, where it does not exist, we should carefully abstain from adding to the injury, by dividing the scalp and exposing the fracture. Many persons have recovered in whom there was at the same time a wound of the scalp and a fracture and depression of the cranium, although no operation was resorted to: three cases have occurred within my own observation. Mr. Abernethy relates two cases in his work on Injuries of the Head, and other cases have been recorded, where recovery has taken place without the operation; I conceive, therefore, that in forming our diagnosis, we should carefully consider the exact nature of the fractures. First, where the depression is from the centre of a radiated fracture, and is depressed to a point, with wound of the scalp, the bone should be raised; for in such instances inflammation of the *dura mater* is almost sure to follow. Secondly, where the fracture is isolated and the piece depressed, it should be removed. Thirdly, where there is a comminuted fracture, with wound of the scalp and loose pieces of bone, the depressed portions should be raised and the loose pieces removed." 393.

We confess we do not see the reasons why Mr. Banner should specify these as *the* cases for operation so exclusively. We wish we could think the solution of the difficulty so facile.

*Secondary Hæmorrhage.*—Mr. Banner observes.—There is one point which does not appear to be clearly understood: it is with regard to the degree of pressure the brain should be allowed to bear before the trephine is applied for its relief in cases of internal hæmorrhage; we sometimes find patients, who are labouring under symptoms of compression of the brain from effused blood, remain in a state which would scarcely justify the application of the trephine, when, from a sudden aggravation of symptoms, they die. Sir Benjamin Brodie, in his work on Injuries of the Head, asks the question: "Does secondary hæmorrhage ever occur within the cavity of the cranium?" and remarks, "In one case which came under my observation I was led to believe that this actually happened, causing sudden death after three or four days of apparent convalescence." In the case just related of John Hughes, the hæmorrhage was very great, so great as to threaten his immediate death, yet the man *walked* a distance of two miles after the accident, and remained sensible for at least three hours after its infliction. It is possible that in this case the artery, which was completely torn across, did not bleed in the first instance.

We conceive that there cannot be a doubt of the *frequent* occurrence of *gradual* hæmorrhage—and of the occasional occurrence of secondary hæmorrhage within the cranium. We know of no law in the economy opposed to either.

With Mr. Banner's opinions on the *treatment of depressions of bone complicated with wound of the brain*, we conclude. He thinks that the surgeon is called upon to use his art for the elevation of the depressed skull; this case must be considered in a very different light to that where an isolated piece of bone has been driven into the brain; here the extraneous matter being detached, loses its vitality, and the restorative powers of the animal system can the more readily act in dislodging it; whereas in the other instance it must ever remain a source of irritation, unless restored to its natural level; it is true that there are instances recorded where persons have recovered in whom a depression of bone has been allowed, under these circumstances, to remain without being elevated; and there are cases also recorded where recovery has followed the elevation of the bone. If we consider this question according to Nature's laws, we shall conclude that where there is a depression of the skull so as to allow a portion of the bone to enter the brain, (the depressed piece being still attached,) and the brain acting on this fixed point, possibly with sharp spiculæ or rough edges, and which from its connexions is supplied with nutrition, and which cannot have penetrated very deeply into the brain, and again, the readiness with which it can be removed, that this irritating substance should be got rid of; and thus the trephine is here rendered useful, as our object should be to raise the depressed bone with as little additional injury to the brain as possible; by the removal of a portion of bone the subsequent steps in the operation will be facilitated.

### ON THE MOVEABLE CARTILAGINOUS BODIES FOUND IN THE SYNOVIAL AND SEROUS CAVITIES. By JAMES MACARTNEY, M.D., F.R.S.

Dr. Macartney observes:—"It is a very generally received opinion amongst practical surgeons that these substances are formed in the first instance on a peduncle or footstalk, by means of which a vascular union is maintained between them and the surface of the cavities in which they are inclosed, and that it is only by the accidental rupture of this footstalk they are set free. It cannot be doubted that such is the fact in some cases, because these bodies are occasionally found attached to the surface of the joints, the tunica vaginalis, the pleura, and the peritoneum, by peduncles, apparently composed of coagulable lymph, and containing blood-vessels; but there appears to be sufficient evidence to prove that, in other instances, they are from the beginning isolated bodies, and devoid of any vascular connexion with the surrounding parts. I have found them placed in circumstances, and organised interiorly in a manner which could only be accounted for by admitting them to possess an independent power of growth and development. For example, I have frequently met with them in the sheaths of tendons without there being the least appearance of their having been connected with the surrounding tissues. In one instance I found above a hundred of these bodies occupying the sheaths of the flexor tendons of the hand, and of various sizes, some being smaller than the pippins of an apple, and very nearly of the same form, while others were two or three times as large, and more spherical in their figure. They had evidently been deposited in succession, and had increased so much in number and magnitude as to raise up the palm of the hand into a convex form."

We apprehend that the loose bodies found in the sheaths of the tendons and the *cartilaginous* substances discovered in joints are not usually of the same description. The former are probably coagulable lymph rounded by attrition—the latter have been attached longer and more highly organised. Dr. Macartney continues,—

"When these moveable bodies are contained in joints, and are subjected to friction and pressure, they seem to be excited to a more rapid growth, and to change their original structure, by the conversion of the cartilaginous matter into bone. I have found them in the knee joint larger than a mulberry, and as rugged as it on the surface, from the unequal growth of bone; and, by macerating some of them, I have found the deposition of osseous matter to pervade their interior. I have likewise known them to sensibly diminish in a short time when kept perfectly at rest and free from irritation." 428.

We confess our scepticism on this latter point, and do not clearly perceive what Dr. Macartney's *proofs* can be. Whether the following case and the attendant remarks can be considered such, we leave to our readers to determine. For our own parts we confess that they are far from convincing. It is much more likely that the tissues of the joint should inflame and swell, than that the loose body should—much more likely that it should slip into some corner than that it should grow less. But to the case.

"A lady, who is now the most distinguished tragedian on the stage, had one of these loose cartilages formed in her knee joint. For some years its presence could only be ascertained by the effects it occasionally produced. When it slipped in between one of the condyles of the femur and the head of the tibia, the pain felt was acute and overcoming; and on one occasion she fell almost as suddenly as if she had been shot, and the whole joint became immediately inflamed. I directed Goulard's lotion to be *constantly* applied in the manner I have described under the name of water-dressing, and enjoined perfect rest, with an extended position of the limb. In a few days she recovered, with only a feeling of weakness and insecurity in the joint, which gradually went off. An elastic knee-cap was ordered, and in the meantime she wore a bandage, which, impeding her movements, she incautiously left off. The second attack of this kind was less sudden and painful, but attended with more swelling of the joint. It was necessary to use leeches; and when the general tumefaction of the knee had subsided, the loose cartilage could be felt on the external side of the patella. It had grown so large that it could not have been received between the bones, and therefore did not produce the same symptoms as in the previous attack. By strict rest and water-dressing she recovered in about a fortnight, at which time the cartilage had diminished so much as to be scarcely discernible. Afterwards the patient wore an elastic knee-cap for upwards of two years, during which she felt no more annoyance from the complaint; when the knee-cap seeming no longer necessary, it was left off, and I believe she has continued well ever since. There are many cases in which persons have recovered with similar treatment; and in such cases it is generally supposed that the cartilage has been removed by absorption. I have related the previous case because it affords a good example, among others I have seen, of these substances increasing under inflammation, and diminishing (whether by means of the absorbents of the joints or not) when the irritation had been removed—a practical fact of importance in the treatment of cartilaginous deposits in joints." 430.

The following is Dr. Macartney's theory.

"No author has given a satisfactory explanation of the origin of these substances, when they have not been connected with the synovial and serous membranes, nor of their increase and change of structure after that connexion is broken, supposing it had previously existed. It is said that Laennec and Bayle asserted that these bodies were at first deposited on the outside of the synovial capsule, through which they gradually made their way into the cavity of the joint. If such were the fact, it could hardly have escaped the observation of other pathologists; but even if we should admit this account to be correct with respect to the *origin* of the loose cartilages, it would throw no light on their subsequent development, after they had passed into the interior of the joint. It is manifest that they could not enter a joint when they had acquired the magnitude and the structure that we know they frequently possess; the joint in its natural state would not be able to receive them. The most curious part therefore of their history is the fact of their growth and conversion into bone independently of any communication with the surrounding parts by means of blood-vessels. It appears to me necessary to admit that they enjoy a separate vitality, that they are influenced by the circumstances in which they are placed, and that they can imbibe nutriment from the synovial fluid and the vapour of serous cavities. This opinion is corroborated by several analogous facts. For example, I have found a tumor of a spherical form floating about in the cavity of the abdomen, which bore on one side the appearance of having the peduncle by which it had been attached broken, and the ruptured part healed with a depressed cicatrix. I possessed also a specimen of anchylosed hip joint, in which a thin shell of the head of the femur had remained unabsorbed, and lay loose in the joint, probably in consequence of escaping pressure, as a process of bone had passed through a hole in its centre and served to conjoin the remaining portion of the head of the femur with the bottom of the acetabulum. This process of bone seemed to have been the round ligament ossified. I also had in my collection a preparation of an injured elbow joint, in which a portion of the external condyle of the humerus had been entirely broken off, and remained loose in the joint. The impression on my mind is, that it was also perfectly separated from all connexion with the soft parietes of the joint; and yet it had acquired a cartilaginous surface from rubbing against the ends of the other bones. This preparation, however, with those that illustrate all the pathological facts I have mentioned, may now be consulted in the University of Cambridge.

The presence of synovia, and the friction and pressure that parts sustain in joints, seem to produce similar effects on different tissues. Portions of lymph, if shed into joints, soon assume the peculiar structure that characterises what are called the loose cartilages. When the ends of lacerated tendons project into joints, a number of knobs or hard bulbs are formed exactly of the same structure, and even nerves, passing naturally among tendons and in places where they receive pressure, assume the same hard dense nature, as may be seen where the median nerve enters the palm of the hand, and where the posterior tibial divides into the two plantar nerves. That very singular change which takes place on the ends of bones, when deprived of their natural cartilages, might as reasonably be supposed to come from a deposit from the synovia, as the secretion of ossific matter of such extraordinary density. I had a specimen of the shoulder joint, in which this porcelanous or vitreous deposit had commenced, and appeared as if the surface was covered by a number of very minute portiôns of glass.

The different kinds and degrees of vitality which may exist in different isolated parts of the animal body constitute a subject of great interest and curiosity, which has not yet been fully investigated. The contents of various cysts seem to possess a distinct kind of existence, and undergo some changes in their interior arrangement. If my memory serve me, there is a preparation in the Hunterian Collection of an extra uterine fœtus, which had been retained in a peritoneal cyst during many years, being converted in a considerable degree into bone. The most striking example of vitality existing in detached parts is displayed in the development of the egg during incubation, when new membranes, new organs, and all the parts of the perfect animal are spontaneously created out of a simple fluid, the albumen merely requiring the presence of the temperature that belongs to the interior of the perfect animal; for it would be a great error to suppose that heat could impart vital power, as, under the same condition, the unimpregnated egg undergoes the putrefactive fermentation.

The vitality of the blood itself, of which no sound physiologist can entertain a doubt, exists independently of any mechanical connexion with the solids of the body; and many of the actions which prove its vital nature are exhibited when it is withdrawn from its vessels, in which it might have received some sensitive influences. It seems not unreasonable to ascribe a degree of vitality to the variolous and vaccine virus, and indeed to all morbid poisons, even in a dried state, as their power of infecting is destroyed by some means which would affect their animal organization, but not necessarily alter their chemical elements. I am aware that some chemical physiologists will deny this; but I would ask such, what is the chemical difference between the fluids of the impregnated and unimpregnated egg? In fine, to determine the precise boundary where vital power ceases and the laws of unorganic matter commence, seems to be hardly possible, without more inquiry and experiment than have yet been applied to the subject; but I think we must admit that there are gradations in vital power, from the highest to the lowest, not only in the different orders of animals, but in the different solids and fluids entering into the composition of each individual." 434.

The quotation is a long one, but less would not do justice to the Doctor's views. A *fair* criticism on them must be longer still, and we cannot in conscience inflict *that* upon our readers. At the same time we must confess, that we think Dr. Macartney pushes his notions of "vitality" too far, or rather, he attributes to the vitality of a part too much of what belongs to the vitality of the whole. We leave, however, Dr. Marcartney and our readers to settle it.

### A CASE OF AMPUTATION OF THE GREATER PART OF THE LOWER JAW. By A. T. S. DODD, Esq., Surgeon to the Infirmary, Chichester.

We notice this case in consequence of a peculiarity in the mode of operating.

Elizabeth Goble, aged 31, married, admitted into the Chichester Infirmary, March 5, 1839. About nine years ago she found a small immovable tumour on the middle of the left side of the lower jaw. When she first noticed it, it was about the size of a nut, not tender nor painful; it has gone on gradually increasing up to the present time, but has grown much more rapidly during the last five years than it did before. Five years ago it was about the size of a walnut; during the last six months it has spread from the symphysis to the submental foramen of the right side of the lower jaw, appearing just in front of the left ear, and reaching round to the right submental foramen; it is lobulated, or rather encysted, and appears to contain a considerable quantity of fluid; she has no pain or tenderness in any part. She had lost several of the teeth of this side three or four years before she found the little swelling first mentioned. The last molar is now bent nearly horizontally inwards by the disease, and the two left incisors are lifted from the sockets, and pushed towards the right side. The healthy structure of the jaw can be felt only on a small part of the ascending ramus and angle; all the rest is enlarged and fluctuating, and may be handled any where without pain, and even the teeth may be removed without suffering. She can open and shut the mouth without any great inconvenience, and can masticate tolerably well with the right side of the jaw. The swelling projected forward so much as to separate the lip from the teeth considerably, and was here, in the situation of the external gum, covered by so thin an integument as to assume somewhat the appearance of a ranula but for its exact situation. This part of the tumour, on being punctured, yielded a straw-coloured, viscid, transparent fluid, evidently contained in a separate cyst, as the puncture emptied only a small part of the tumor. The integument of the cheek was perfectly sound; health good; catamenia regular. It was now evident that the disease consisted of encysted cavities, occupying and destroying the structure of the whole left side of the jaw; that the upper jaw was not implicated; that there were no enlarged glands in the neighbourhood; that the health was good, and the boundaries of the disease well defined.

Mr. Dodd determined to remove the disease, preserving as much of the jaw as possible. The following is the description of the operation:—

"The patient being seated in a chair, and the head supported, an incision was made from nearly opposite the articulation of the jaw to the angle; from thence along the horizontal ramus to the symphysis. The flap of the cheek thus formed, was then reflected upwards over the tumour; the mouth was then opened at the junction of the gum with the jaws, opposite the canine tooth; the gum was freely divided from before to behind, and the entire cheek turned back; in like manner the tongue was separated from the jaw, by division of the inner gum from about an inch to the left of the symphysis, as far back as the angle. The jaw was now divided through the mass of disease in the situation of the left canine tooth, partly by the knife, and partly by shears: but the bony structure was so attenuated by pressure of the cysts, that it was not thicker than cardboard, and was therefore readily cut through. Now taking the horizontal branch of the jaw in my hand, and having with the other separated the tongue from it as far back as the coronoid process, I endeavoured, by depressing the jaw upon the neck of the patient, to bring the process forward, that I might the more readily divide the temporal muscle at its insertion. I found however that the attenuated bone was so flexible throughout, that this was impossible; I therefore cut upon the coronoid process, which was enlarged and occupied by a cyst to the very tip; it readily yielded to the knife, being only of the consistence of tough membranes; I divided it across, and afterwards with the scissors and forceps removed the lip from under the zygomatic arch. The flexible state of the ascending ramus also prevented my throwing the head of the bone forwards from the glenoid cavity as I intended; I therefore cut down to the anterior part of the articulation, and then, fearing to pass my knife round the head of the bone from before to behind, in the immediate neighbourhood of the large vessels, I lifted the whole portion of the jaw up, and, drawing it steadily forwards and upwards, I divided from behind the pterygoid muscles, and then opening the articulation at the back part, readily passed the knife through it, from behind forwards, and thus completed the dissection. I now tied the facial artery, which was the only one that then bled, and proceeded to remove the diseased symphysis; this was readily done by dissecting off the integuments in front, dividing the muscles of the tongue behind, and then sawing through the bone near the right submental foramen. The canine and lateral incisor teeth of this (the right) side had been extracted before the operation commenced. The patient bore the operation, which lasted about forty-five minutes, with remarkable fortitude. She lost very little blood; several arteries gushed out on their division, and one large vein while opening the articulation; but the hæmorrhage was commanded by pressure of the finger at the time; and after she was put to bed only two ligatures more were required. Eight sutures were applied in the course of the wound, about four hours after she was put to bed, and the parts were supported with a compress and plaister." 439.

The patient did perfectly well, and we need not pursue the after details. We quote Mr. Dodd's observations on the mode of operating he adopted.

"It was necessity, and not my own choice, which led me to adopt the plan of opening the joint from behind; I intended, in planning the operation, to follow the mode recommended by Mr. Liston. The very flexible state of the ascending ramus prevented however any attempt at throwing the condyle forward; but I found the plan of drawing the whole portion of the jaw forwards and upwards, and passing the knife from behind to before, so very feasible, and, as it appears to me, so much more likely to escape the danger of wounding the great vessels in the neighbourhood than by disarticulating from the front, that I confess I am rather at a loss to know why preference is given to this latter method by so experienced a surgeon as Mr. Liston. As it was, I found that the knife actually exposed the sheath of the carotid vessels; and it appears to me to be much more easy to avoid them by dissecting from below upwards, with the jaw drawn forwards, than by turning the knife round the head of the bone from the front of the joint, and so, in passing it downwards, separating the ramus from these important vessels.

Thirdly, I divided the jaw in two places: first at the situation of the left submental foramen, through the disease itself; and afterwards at the same point on the right side, thus making two stages of the operation, and removing the symphysis after the disarticulation of the jaw had been accomplished. This proceeding I adopted in consequence of the caution given by Mr. Liston (whom I consider the highest authority we possess on this subject) against the danger of the retraction of the tongue upon the division of the muscles which attach it to the symphysis of the jaw. He considers that there is more danger and embarrassment to be expected from this part of the operation than from any other. The detachment of the anterior belly of the digastric, of the genio-hyoid, and genio-hyo-glossi muscles is apt to be followed, unless great precautions are taken, by retraction of the os hyoides and tongue, and a sudden interruption of respiration. With these dangers in view, I avoided them till the very last, by following the plan above mentioned, and thus leaving the division of the muscles of the tongue till the very last stage of the operation. By this plan the operation was somewhat lengthened, but it was rendered more secure. I must however add that when the muscles in question were divided, no retraction of the tongue took place, nor had we any difficulty of the kind afterwards. Whether such accident would have occurred if the muscles had been cut through in the early stage of the operation, and the tongue had been thus left unsupported during the whole of this trying period, cannot now be decided certainly; at any rate it is more than probable, and I consider myself quite borne out in the proceeding which I adopted by the experience of so eminent a surgeon as Mr. Liston, and even by the avoidance of a probable danger so important as that in question." 443.

Mr. Dodd ranks high as an operating surgeon, and this case redounds to his credit.

Cases of Abscess forming within the Pelvis after Labour, with Observations. By Thomas W. Wainwright, Esq. Surgeon to the Liverpool Northern Hospital.

Mr. Wainwright observes that his attention was drawn to this subject many years ago by the late Henry Park. His observation was as follows:—" In the course of my practice I have met with several instances of abscess forming in the cellular texture of the pelvis after labour; the thigh becomes flexed upon the pelvis, and the matter at length is discharged, sometimes at the groin, and sometimes near the anus, and at others above the spine of the ilium. They are very distressing cases, and seem almost hopeless; but the great majority of them, notwithstanding, get well. I am sure the subject deserves more attention from the profession than it has yet received, and I hope some one will give it."

Mr. Wainwright relates seven cases. Three will sufficiently illustrate, we think, the affection.

*Case* 1.—Mrs. W., æt. 24, was confined of her first child February 8th, 1835; nothing particular occurred during the labour; but a few days afterwards she became, as it is termed, " excessively nervous." She took a great dislike to her nurse, and at length could not endure her presence. Six weeks passed in this way, and her nervous symptoms began to subside. Then she complained of severe pain in the right groin, extending along the fore part of the thigh; in two weeks more the thigh became flexed on the pelvis nearly at a right angle, the bowels were much constipated, she had copious perspirations, and became much emaciated. In this state she was conveyed to Liverpool in April. The tenderness in the groin was excessive, and she would scarcely allow the slightest examination; a tumour however was easily felt, deeply seated between the groin and pubis; the motions were observed at this time to be almost white. Blue-pill and extract of henbane were given at night, mild aperient injections were administered each morning, and the diet strictly attended to; depletion was not considered desirable in her debilitated state. The secretions improved under this treatment; the pain was also relieved, and the thigh became less flexed, and the tumefaction remained as before.

The patient now resolved to go to the Isle of Man, where a temporary improvement took place; but the symptoms soon became worse, and she returned to Liverpool in June. She was then in a distressing state; more emaciated from constant pain and frequent perspiration; the swelling greatly enlarged, and, having advanced to the median line, it felt in form exactly like the distended bladder. She complained of a sense of fulness at the anus; and on examination, *per anum*, the cavity of the pelvis appeared filled up with some kind of tumor. Next morning "something" passed with the urine "all at once." It was a large quantity of pus. The tumor was much diminished in size, and she was greatly relieved. This occurred on the 30th of June. Matter continued to flow involuntarily from the vagina for about a fortnight, whilst the urine passed unmixed as usual, so that it was evident an abscess had opened into the vagina. The limb gradually recovered itself, her general health rallied, and in siz weeks from the bursting of the abscess the catamenia reappeared for the first time since her labour. She was again confined in July, 1837, and also in April, 1839, recovering each time remarkably well.

*Case* 2.—Mrs. R. æt. 29, was confined of her first child January 14th, 1839; the labour lasted twenty-four hours, and was severe; the child was large and was born dead; the placenta separated naturally. All appeared likely to do well during the greater part of the first three days; the secretion of milk was abundant, but the lochia rather deficient in quantity, and somewhat purulent in appearance, but without any offensive odour. On the third day she had rigor, and this was followed by fever, pain in the head, occasional delirium, diminution of the above secretions, and pain in the left iliac region. Venæsection and leeches were employed with relief to the general symptoms, but the pain in the iliac region remained as before and resisted every treatment. She soon experienced great difficulty of motion; if she attempted to walk the body was bent forward, and every effort to place herself in an erect position caused great suffering; but the thigh was not obliged to be kept constantly flexed on the pelvis. About eight weeks after her confinement she perceived a uniform swelling above Poupart's ligament; this gradually increased, and was accompanied with a sense of numbness of the whole limb, but no swelling of it. A fortnight after this the tumor was opened, and a large bleeding bason full of matter was evacuated. She was now extremely reduced in strength, and greatly emaciated; had profuse perspirations, and had suffered several attacks of diarrhœa. The discharge was very copious for some time, but gradually ceased in about six weeks. Her health began to improve from the period when the matter was evacuated, and has since remained very good.

The remaining case that we shall insert was a fatal one.

*Case* 3.—Ellen Clements, æt. 22. May 21, 1835.—This patient is in a very emaciated state. She has a fulness in the right iliac region, which is painful on pressure, and the pain extends towards the lumbar region; the thigh is somewhat flexed on the pelvis. She has a discharge from the vagina, like healthy pus in appearance, but horribly offensive in smell. By the use of the speculum this matter is seen to flow through the os uteri. It appears that about two months ago she was confined of her second child. The labour was natural, and of short duration; but the accoucheur informed her that a portion of the placenta remained, and would ere long come away. Ever since the labour an extremely offensive discharge has continued to pass from the vagina. Leeches, blisters, &c., were applied to the iliac region, and a weak solution of chloride of soda, in warm water, was used as an injection, at first into the vagina only, but afterwards into the cavity of the uterus. The vaginal discharge seemed checked, but it was soon discovered that the pain and the iliac tumor were increased in proportion; and, on the other hand, that when the discharge was more copious from the vagina, the tumor and her sufferings were diminished together; the injection was therefore abandoned. She gradually became weaker and weaker, her appetite failed, and she died July 10th. Mr. Long diagnosed that abscess existed in the iliac fossa, and that a communication would be found within the cavity of the uterus.

*On dissection,* a large collection of dark-coloured pus lay beneath the peritoneum directly upon and below the iliacus internus muscle; its investing fascia was almost entirely destroyed; the muscle itself was of a dark, almost-black colour. *Upwards,* the matter had passed along the psoas muscle, as far as the third lumbar vertebra, laying bare the two inferior transverse processes. *Downwards,* it had got among the muscles on the upper and fore-part of the thigh; it encircled the hip-joint, and had penetrated the capsular ligament. The cartilages, synovial membrane, and ligamentum teres, were nearly destroyed. The matter also passed round to the dorsum of the ilium, from thence over the crest, and thus communicated with the abscess in the venter; the periosteum covering both sides of the ilium was as easily detached as from a bone subjected to maceration. *Inwards,* the matter had completely dissected the anterior crural nerve, psoas muscle, and the crural vessels; and beneath these it had passed into an irregular filthy cavity, one inch and a half in diameter, formed by the uterus and the side of the pelvis. From this cavity, a round opening, sufficient to admit a crow quill, penetrated the uterus about three-quarters of an inch above the os tincæ, and conveyed the matter into the cavity of the uterus, and from thence it had passed into the vagina. The nerves were firm in texture, though brown in colour, being bathed in dark-coloured pus. The external iliac artery and vein were firmly united together, and surrounded by a brown substance, probably the cellular tissue infiltrated with pus. The uterus was of a dark venous colour, but of the natural size. The inner surface, near the fundus, presented a small white spot, to which it was supposed the placenta had adhered. The right sacro-iliac synchondrosis was separated sufficiently to admit a sixpence, and also to allow a slight motion; but no appearance of inflammation or of suppuration could be seen at this point. The other synchondrosis and the symphysis pubis were carefully examined and found healthy.

These cases are worthy the attention of our readers.

### Cases illustrative of the Pathology of Biliary Concretions. By George Mallett, Esq. Surgeon, Bolton-le-Moors.

Mr. Mallett relates three interesting cases of gall-stones. We are sorry that we have not room for more than a notice of the post-mortem examination of one. It shews a gall-stone imbedded in the pancreas.

There was an ulcerated opening through the coats of the gall-bladder, communicating with an abscess beneath the concave surface of the liver, which contained about six ounces of pus. The ulceration was evidently caused by the irritation of a moderate-sized gall stone, which was found near the opening, but still within the bladder. Upon examining the other organs, all were found in their natural condition with the exception of the pancreas, which contained a hard encysted body, and which proved to be a gall-stone about three-quarters of an inch in diameter. The pancreas was not found either in appearance or structure altered; and the concretion must have been imbedded there for some time, as no trace of its passage could be discovered. Thus, then, was explained the cause of his sufferings some years previously, and their cessation till another gall-stone had formed, and then expulsion in a similar way was attempted; but inflammation, abscess, and death, were the consequences.

### A Report of the Out-Patients attended by F. Ryland, Esq., at the Birmingham Town Infirmary, between the 25th Dec. 1837, and the 12th Dec. 1839.

We regret the following announcement:

"The present report contains the result of parochial practice for nearly two years, viz., from the 25th of December, 1837, to the 12th of December, 1839; at which latter period I resigned the appointment of Surgeon to the Birmingham Town Infirmary." 481.

Mr. Ryland has placed the tabular reports for two years side by side, in order to shew the great resemblance that one year bears to another in the numbers that are attacked by such diseases as owe their origin to the season, to occupation, habits of life, or accidental circumstances, and the disparity which exists between the numbers affected in each year by the epidemic and contagious diseases. Hooping-cough, which at first sight appears to offer an exception to this remark, does not do so in reality; for though the numbers in each year attacked by this disease approach to an equality, this is owing to our having in the table the rise and declension of a single epidemic, which commenced in the second quarter of 1838, continued through the autumn and winter, and disappeared in the summer quarter of 1839. In 1838, there were fifty-two cases of small-pox; in 1839, only three; whilst of scarlatina, in 1838, there were three cases only, and in the following year thirty-two.

The cases of rubeola were, for the whole six years, two hundred and two in number, and the deaths thirty-six, being about one in five and a half. The number of cases of scarlatina was two hundred and thirty-eight; of deaths, thirty-three, or one in seven and a quarter. The cases of variola numbered one hundred and seventy-nine; the deaths from this disease were thirty-six, or one in five.

It appears therefore that scarlatina occurs rather more frequently in Birmingham than measles, and the latter more frequently than small-pox; and that the rate of mortality of these three diseases is in an inverse ratio with their frequency. The prevalence of measles as an epidemic, in 1834 and 1836, is well shown in the table. The moderate continuance and harmless nature of scarlatina in 1834 contrast well with its great prevalence and increased mortality in the latter part of 1835 and in the beginning of 1836. The same disease is seen to have occurred in a sporadic form in 1837 and 1838, and to have again become epidemic towards the close of 1839. It has since produced great ravages in Birmingham. Variola was prevalent and very fatal in the first half of 1834, after which it subsided for several months; it re-appeared at the close of 1835, and continued, with moderate frequency, till the end of 1838, since which time there have been scarcely any cases of this disease.

The *measles* of the latter part of 1839, though the cases were neither numerous nor fatal, was marked by some peculiar features, which were attributable in all probability to the long continuance of cold and damp weather during the summer and autumn of that year. The cases, thirty in number, all occurred in September, October, and November. The earlier cases were simple in their nature, and soon recovered; but about the middle of October it was observed that the catarrhal symptoms attendant upon the attack of measles were of an especially severe nature. The secretion from the nose was great and most acrid in its character, occasionally mixed with blood, and causing much excoriation in the neighbourhood of the nostrils. The mucous membrane of the mouth also inflamed and ulcerated, and the tonsils partook largely of the inflammation; the submaxillary and cervical glands became swelled in some instances to such a degree as seriously to impede respiration and deglutition. In two of the fatal cases the measles were complicated with asthenic bronchitis, which however appeared to yield to stimulating expectorants, counter irritation, and good food, but the mucous membrane of the mouth, in both instances, became extensively ulcerated, the gums separated from the teeth and from the alveolar processes; the submaxillary glands and the surrounding tissues swelled so much as to render the chin scarcely discernible, and death took place about the fourteenth day. The other fatal case was similar in its result, but much more rapid in its course. The patient, a boy, æt. 2, broke out with measles on the 2nd of November. The eruption was well marked, and the catarrhal symptoms severe. On the 4th there was acrid discharge from the nostrils, with aphthous ulcerations about the inside of the lips and edges of the tongue. The submaxillary glands were enlarged to such a degree that the neck was almost continuous with the cheeks, and the chin was undiscoverable; respiration was slightly impeded, hard cough, and great difficulty of swallowing. The child died on the 5th, (the fourth day of the disease,) apparently exhausted, without evincing any sign of suffocation. Permission to examine the body could not be obtained.

The glandular swellings generally yield to the application of leeches, fomentations, and poultices; in two or three instances they terminated in abscess. In the scarlatina of the present winter similar glandular swellings have been present in almost every case.

Two of the cases of measles were complicated with inflammation of the trachea. In the first, a stout lad between two and three years old, the eruption was preceded more than two days by severe croupal symptoms, which required bleeding and strong doses of calomel and tartarised antimony for their removal. When the measles were fully developed, the croupal affection became much better; but as the boy had taken in the two days more that fifty grains of calomel and five grains of tartarized antimony, some of the amelioration that took place may safely be ascribed to the medicine. Mr. Ryland, however, has often noticed in these cases that the appearance of the rubeolar eruption is followed by amendment in the croupal symptoms. This boy was convalescent on the seventh day, and soon recovered completely. The other case was a girl, seven years of age, residing in the same court. The measles and the tracheitis commenced together after slight precursory illness of seven days' duration; the full development of the eruption was neither beneficial nor otherwise to the croupal attack in this case. Leeches applied along the sides of the trachea, and small doses of calomel and antimony soon relieved the urgent symptoms; general bronchitis succeeded to the tracheitis, and the girl ultimately got completely well.

*Scarlatina* was rather prevalent during the last quarter of 1839; but, relatively considered, it was not very fatal in its effects. Three instances of death from this disease are recorded; one from malignant cynanche in combination with scarlatina; the second from head affection, and the third from a cachectic condition induced by the fever; the cornea of the left eye sloughed, the umbilicus was implicated in a large sloughy ulceration, the nates also sloughed, and the child died between three and four weeks from the time of seizure.

*Fever* was not so prevalent in 1839 as in the previous year, nor was it quite so fatal in its effects: its character was in many instances, and particularly in certain localities, typhoid, and two of the children who died of this disease had gangrenous inflammation of the mouth and necrosis of the upper jaw.

Inflammatory affections of the mucous and serous membranes were less frequent in their occurrence in 1839 than in the preceding year, and both absolutely and relatively were less fatal.

The deaths from carcinoma, phthisis, and scrofula, in 1838, were seventeen, bearing a proportion to the whole number of deaths in that year of about one to five. In 1839, the deaths from the same three diseases amounted to twenty-five, which was more than one-third of the total number of deaths for that year. Of the forty-two deaths from these causes, twenty were males, all of whom died of phthisis. Of the twenty-two females, fourteen died of phthisis, six of carcinoma of the uterus and mamma, and two of scrofula.

Some other remarks and several tables will repay perusal.

A REPORT OF THE CASES ATTENDED AT THE BIRMINGHAM EYE INFIRMARY, During the Years 1838 and 1839. By RICHARD MIDDLEMORE, Esq., Surgeon to the Infirmary.

1. *Case in which an Operation for Artificial Pupil was performed on an Eye previously affected with Staphyloma of the Cornea.*

"In the sixth volume of the *Transactions* I related the case of James Shephard, who became my patient at the Eye Infirmary, after having left the Birmingham Hospital, into which he had been admitted in consequence of a severe accident which had seriously injured his face and eyes. When I first saw him, the *right* eye was entirely collapsed. More than half the lower portion of the *left* cornea was extremely opaque and prominently staphylomatous, the pupil was almost entirely obliterated, and the iris extensively adherent to the upper margin of the staphyloma. The mode of treatment employed for the cure of the staphyloma consisted in the repeated tapping of the part by means of a fine iris-knife; it being evident that excision or any similar mode of cure would have unfitted the eye for the performance of an operation for artificial pupil, which presented the only chance of restoring to the patient any degree of vision. On the cure of the staphyloma, the patient being still blind, was persuaded to go to the Bristol Asylum, where he was taught to make baskets. He remained there until June, 1839, when he returned to Birmingham, and called upon me, expressing a strong wish to have an operation performed. On this occasion he was led to my house by a little child, being unable to find his way, without assistance, about the streets of the town in which he had lived many years." 501.

At this time *more than one-half* of the cornea, at its lower part, was densely opaque, the pupil nearly obliterated, the iris extensively adherent to the opaque cornea; but, from the slight glance of an exceedingly minute pupil which it was just possible to obtain, by looking downwards as the patient sat on a low seat, it was ascertained that the lens and its capsule were transparent.

"*Operation.*—August 6th, 1839. Present Mr. Jones and Mr. Clarke.—Raising the upper lid without pressing upon the eye-ball, I made a small section of the cornea at its superior part, through which I introduced a fine iris hook, and very cautiously drew out a portion of iris, which, as I could not excise it with the curved scissors without a risk of touching the capsule or dislocating the lens, I left to be strangulated between the lips of the incision of the cornea. The iris bled a good deal after the operation; but by keeping the patient quiet in bed, and by the adoption of various antiphlogistic measures, the effused blood was absorbed, acute inflammation was prevented, and the patient's vision so far restored that he has this day read to me, with tolerable ease, and without glasses, a portion of the former part of his case published in the sixth volume of the *Transactions.* The pupil I have made has a smooth margin, being formed, not by excising a portion of iris, but by detaching its pupillary margin from the staphymatous cornea, to which it was extensively adherent; it is of good size, being neither very large nor unusually small; it is somewhat oval in shape, and extends from the upper border of the staphyloma nearly to the corneal margin at its superior part, where I made the incision."

Mr. Middlemore has received and merits great praise for this proceeding.

2. *Ossification of the Lens.*

The patient is David Phillips, a silk dyer, aged 45. About sixteen years ago he received a blow upon the left eye from a piece of turnip, which was purposely thrown at him. The eye was a good deal inflamed soon afterwards, and required treatment, which had the effect of relieving, and, as he then thought, of curing him. About a year afterwards his friends perceived a *speck* in the eye; at this time his vision was impaired, and was soon entirely lost. Lately the sight of the *right* eye has become dim, and on this account, as well as on account of the state of the *left* eye, he came to the Infirmary.

*State of the right eye.*—Pupil rather larger than natural; movements of the iris sluggish; vision by no means perfect.

*State of the left eye.*—A solid body is situated in front of the iris, in which are seen a number of densely white lines, radiating from its centre to its circumference. It has the shape and size of the crystalline lens. The eye is scarcely at all inflamed, but he says it feels very uncomfortable.

With Beer's knife, Mr. Middlemore divided the lower part of the cornea quite as freely as in an ordinary operation of extraction, and in a few minutes, on making slight pressure upon the eye-ball, the ossified lens passed through the opening. In a fortnight the patient was able to return home. The pupil is circular and clear, the wound united, and vision slightly improved. The lens is ossified throughout the whole of its texture, it is of its ordinary size and shape, has a radiated appearance, and is of a dirty white color.

3. *Case in which one Eye was extremely Large from Birth.*

James Bembridge, æt. 4. Left eye of ordinary size and dark colour.

*Right eye.*—The cornea is nearly double the size of that of the left eye; the anterior chamber amazingly ample, so as to equal in size an extreme case of dropsy of the anterior chamber. The pupil is large, and sluggish in its action. The cavity containing the vitreous humour is as large in proportion as that containing the aqueous humour. At the upper part of the cornea, and along its margin, there is a peculiar nebulous appearance, as though the cornea was seen through the conjunctiva, which was prolonged upon its surface, so as somewhat to overlap its margin. The boy has scarcely any sight with this eye; it is however free from inflammation, and by no means very painful. The lids are projected by the large globe; sometimes the right eye is rendered a little uncomfortable, and the appearance of the child is peculiarly disagreeable.

"I have lately," observes Mr. M. "seen a similar state of eye in a patient attended by Mr. Royston, of Redditch. In this, as well as in Bembridge's case, I have recommended that nothing should be done unless the globe should continue to enlarge, unless it should give rise to irritation of the opposite eye, or unless the subject of the affection should after a time be anxious for an operation on the ground of personal appearance. I have in several such instances evacuated the aqueous humour with a fine cataract needle or iris knife, and in this way prevented the disease from increasing. In one instance I excised a small portion of the centre of the cornea, and with the effect of much lessening the size of the globe, but not of producing its diminution sufficiently to allow of the patient's wearing an artificial eye." 506.

4. *Tumor situated at the Cornea-Sclerotic Junction.*

Mr. Middlemore relates two cases. We shall insert one.

*Case.*—" William Davis, æt. 25, has a small tumor, about half the size of a pea, situated partly upon the cornea and partly upon the sclerotica, or to speak more correctly, it appears to supersede a portion of the membranes, whose place and situation it occupies. It is of a dirty white colour, convex externally, of a firm texture, and apparently covered by conjunctiva, which cannot however be moved upon its surface. The tumor has existed from birth, and does not, he says, increase in size. He came to me, at the infirmary, in consequence of the irritation produced by a few hairs which grew from a slight depression at the centre of the little tumor. These hairs are curved like the eye-lashes, are of the same colour, about the same length, but are scarcely so strong. The presence of the hairs was first pointed out to him by a friend about a year ago; and even now they excite scarcely any irritation, except when the longest of them reposes on the cornea. On the extraction of the hairs all irritation subsided. I have requested him to procure a proper pair of forceps, and to remove them for himself whenever they are sufficiently long to occasion him any inconvenience." 507.

Mr. Middlemore remarks :—" This is an extremely unusual defect. I have many times seen a tumor similar to that in the eye of Wm. Davis, (whose case has been just related,) which is of a dirty-white colour ; and I have also seen tumors of a red colour, and possessing a granular surface, somewhat like a small mulberry ; but it has rarely fallen to my lot to notice a disease such as exists in this instance. And here I may remark that in no instance which has fallen under my observation has the disease existed in both eyes. I believe that in cases like the present we had better not interfere, at least with a view of removing the mere personal defect about which parents are generally very anxious. They seldom give rise to much inconvenience, or increase in any material degree. In one instance, somewhat similar to this, an attempt was made by a surgeon to remove the disease by shaving the prominence it occasioned down to the level of surrounding parts, and the result of this improper proceeding was suppuration of the eye-ball."

5. *Large Tumor produced by the Escape of the Vitreous Humour beneath the Conjunctiva.*

Joseph Hill, æt. 19, received a blow upon the eye about six years ago, on account of which he applied to a surgeon, who, after the lapse of several weeks, succeeded in removing the inflammation excited by the injury, but was unable to preserve the sight of the eye. About two months ago he received a slight injury of the same eye, which produced a small swelling, which has rapidly increased until it has acquired its present size.

*State of the eye.*—Cornea slightly opaque, particularly at its temporal side; pupil closed. At the outer side of the cornea there is a tumor as large as a good-sized walnut, which evidently contains fluid.

Mr. M. introduced a fine cataract needle into the swelling, discharged a quantity of thin pale yellowish fluid, and then directed the patient to close the lids, upon which he passed a fine roller so as to prevent them from being opened, and requested him to keep the part quiet for a few days, to take a little aperient medicine, and confine himself to low diet. In a few weeks the eye was pretty much in the condition it had been in previously to the last injury.

6. A curious case is related, in which *a piece of tobacco-pipe, one inch and three-eighths long,* was impacted in the orbit, and sneezed out. Vision of the eye was lost, although the eye-ball was not wounded.

7. *Partial Dislocation of the Lens through a Rent in the Sclerotica.* Sarah Hanson, æt. 55, received a blow upon the eye whilst breaking a stick.

*State of the eye two days after the occurrence of the accident.*—Small tumor at the nasal side of the sclerotica, a little behind its junction with the cornea, which is covered by the conjunctiva. The pupil is drawn in the direction of the tumour as though a portion of the iris were absorbed. One part of the margin of the lens is seen to occupy a great portion of the pupil, the other is placed behind the corneo-sclerotic junction, and is covered by the conjunctiva, which is of a dark-red colour, from the effects of the recent contusion.

*Operation.*—Lens readily removed by a free division of the conjunctiva.

*After-treatment.*—A light roller was passed around the lids, which were carefully closed. The patient was directed to keep her bed, and to take a little aperient medicine. No severe inflammation occurred after the operation; and, in the course of a few weeks, there was the following entry—" *Cornea* clear, *pupil* a little drawn towards the lacerated opening in the sclerotica, *vision* extremely imperfect."

8. *A Piece of Steel in the Anterior Chamber in Contact with the Iris.* "John Pugh, æt. 32, tool-maker. Whilst at work, a portion of hard steel was detached from a punch, and forcibly struck his left eye. Two days afterwards he called upon me, when I made the following notes of his case :—" Pupil clear, but contracted ; its mobility somewhat impaired ; iris dull and in a state of inflammation ; at its temporal side and in its anterior surface a small portion of metallic-looking substance may be perceived, which he tells me is a portion of hard steel detached from a punch with which he was working."

*Treatment.*—Bleeding, mercury to the production of moderate ptyalism, cooling lotions, and low diet.

*Result.*—After remaining on the books little more than a fortnight he was discharged, his eye being quite well, with directions to return if a relapse of inflammation should take place.

The metal is still to be seen in contact with the iris, but his vision is as good as it was prior to the accident. The only difference of any sort which can be perceived is, that when the pupil is expanded that portion of the iris with which the metal is in contact (the anterior surface and temporal side of the iris) appears to be paralysed, so that the pupil possesses a shape very much like the letter D.

This is another instance exhibiting the good effects of making no effort to remove a foreign body by a surgical operation, when it has completely passed into the anterior chamber.

### Suggestions as to a Form of Register for Hospitals, Dispensaries, and Private Practice. By Charles Cowan, M.D.

We quite agree with Dr. Cowan on the value, nay necessity, of an accurate registry of cases. Medicine would be greatly improved were such general and uniform in private practice as in public.

Dr. Cowan suggests that the size of the register, when open, should not be less than from thirty to thirty-two inches across, and the depth of the page about twelve. This, including the printed headings on the top, will admit of the insertion of eighteen cases on lines six-tenths of an inch apart. The paper should be strong, and ruled in red ink, and the book thick in proportion to the wants of the institution, and adapted for at least two years' registration, to avoid expense. Separate books should be provided for the physicians and surgeons, and also for the in and out-patients. If two physicians or surgeons attend on the same day, each officer ought to have his own book. The cases should be registered invariably at the time of admission, and every thing inserted which can be then ascertained. In dispensaries of limited extent, a single register might prove sufficient.

We shall now make a few remarks on the use of the register.

A simple inspection will show that many of the columns require only a mark (1), as those for *age, sex, married, single, recovered, made in or out-patient*, and they follow each other in a convenient and natural succession. The "*number of days on books*" is the interval between the date of admission and date of discharge; and the "*total duration*" consists of the addition of the two columns immediately preceding it. Except when the diagnosis is not doubtful, it is better to insert it when the patient is discharged. The usual single column of "*result*" has, it will be seen, been divided into several, for the purpose of accuracy and easy revision. "*Incurable*" includes all cases evidently hopeless; these may be also marked as "*relieved*" or "*left sick*," if not benefitted. "*Convalescent*" should be restricted to such cases as are fully expected to recover. "*Irregular*" includes all cases where the result is unknown. "*Cause of death*" is intended for instances where the fatal result is depending on accidental causes; the use of it is to make the estimate of the mortality more accurate. "*Made in or out-patient*" is an important point to notice, the same patients being frequently counted twice over in reports. "*Treatment*:" this column is optional, but should always contain a statement of any surgical operation. "*Observations*:" this is also optional, but may be used to give an outline of the case, or to refer to any particular point of interest; or the space may be occupied by any other fact which the observer may wish to notice. The "*state of health*" of the patient at the time of admission would be a useful detail; and there are other points which might be also recorded, but which had better be left to the zeal and sagacity of individuals.

Dr. Cowan also suggests a common nomenclature for disease—and he recommends that of Mr. Farr.

*The following are the headings we recommend, with the spaces of course proportioned to the nature of the fact, and the size of the book :—*

RIGHT HAND SIDE.

| No. | Name. | Age. | Sex. M. | Sex. F. | Married. | Single. | Residence. | Occupation. | Date of admission. | Date of discharge. | No. of days on books. | Anterior duration. | Total duration. | Disease. |
|---|---|---|---|---|---|---|---|---|---|---|---|---|---|---|
| 1 | John Cooper | 20 | 1 | .. | .. | 1 | Reading | Smith. | Jan. 1 | June 21 | 20 | 10 | 30 | Bronchitis. |

LEFT HAND SIDE.

| Recovered. | Relieved. | Convalescent. | Left sick. | Incurable. | Irregular. | Died. | Cause of death. | Made in or out-patient. | Treatment. | Observations. | Medical attendant. |
|---|---|---|---|---|---|---|---|---|---|---|---|
| .. | .. | 1 | .. | .. | .. | .. | .......... | 1 | *Mercury, local depletion* | ............. | *Dr. Cowan* |

" Before concluding this part of our subject we would submit the following form of headings for cases, which is rather more convenient than the one we previously proposed. The remainder of the sheet is to be divided by a central line, one side for observations, the other for treatment. When bound, they form a convenient case book, and when separate are well adapted for dispensary practice. Patients attending the institution should leave them on the spot, and if seen at home should always have them ready for the medical attendant. If kept distinct for each medical officer, and filed as the patient is discharged, no confusion would result. The convenience of the practice is great, saving trouble and assisting the memory.

| | | | |
|---|---|---|---|
| Name | No. | Age | State |
| Occupation | Residence | | |
| Anterior duration | Admitted | Discharged | Days treated |
| Cause | Hereditary | Result | |
| Diagnosis | | | |
| Observations | | | |

This register may with very slight alterations, be adapted for private use. For this purpose it is only necessary to devote the left hand page, after the last column for result, to " treatment" and " observations."

